# Supplementary material for: Gestational Dysfunction-Driven Diets and Probiotic Supplementation Correlate with the Profile of Allergen-Specific Antibodies in the Serum of Allergy Sufferers
Source: Nutrients. 2020 Aug 9;12(8):2381. doi: 10.3390/nu12082381 (PMC7468854; doi:10.3390/nu12082381)
Supplement: Supplementary file 1 [file nutrients-12-02381-s001.pdf]

Appendix A1-EuroPrevall Survey on the course of allergies

Case-Control Questionnaire

1. Age: .... years

2. Sex: M/F

FOOD ALLERGY

3. Does the eating any of the following foods causes health problems? If the avoidance of any of them is in progress please put the cross in proper column. If not please provide an approximately how often following foods has been eaten

Table S1 Case-Control Questionnaire

FOOD	Does the eating any of the following foods causes health problems?		Avoidance (If yes)	How often the following food has been eaten Tick one column only			
	Yes	No		Most days	Most weeks	Most months	Never
Cow's milk*							
Hen's eggs							
Fish							
Shrimp, seafood shellfish							
Peanut							
Hazelnut							
Walnut							
Chocolate							
Apple							
Peach							
Bananas							
Kiwi fruit							
Orange							
Tomato							
Potato							
Avocado							
Carrot							
Celery							
Soybean							
Lentils							
Wheat**							
Buckwheat							
Corn							
Rice							
Sesame seed							
Mustard seed							
Sulfur dioxide and sulfites							

*Other cow's milk products including butter, cheese, yoghurt, kefir, crème fraiche, ice-cream, shake

**Including wheat products such as bread and breakfast cereals, bars, pancakes, pastry.

4. Has this child/participant had any problems eating any ***other*** food or foods?

Yes/No

If 'YES': please list that foods:………………………………………………………………………………..

5. Which of these foods mentioned above in points 3 and 4 caused ***the most severe problems***? ……………………………….

5.1 How old was this child/participant/participant when had his/her first problem eating this food?

....

5.2 How often has this child/participant/participant had problem after eating this food?

Only once;
2–4 times;
More than 4 times

5.3. When was the last episode?

……………………..

5.4 Has this child/participant/participant avoided eating this food since his/her illness or problem?

Yes/No

5.5 Does this problem was manifested like: (put the symptom in a circle possible multiple choice)

1- Itching, tingling or swelling in the mouth, lips or throat
2- A rash, nettle sting like rash or itchy skin
3- Diarrhea or vomiting (other than food poisoning)
4- Runny or stuffy nose
5- Red, sore or running eyes
6- Difficulty swallowing
7- Breathlessness
8- Stiffness in his/her joints
9- Fainting or dizziness
10- Headaches
11- Anaphylaxis
12-Other: (if yes please describe) ……………

5.6 How long after eating the food did the first symptom appeared?

-minutes;
-hours;
-days

5.7 How long did it last?

-minutes;
-hours;
-days

5.8 How severe the manifestation was:

Use the 10 points scale where 1-is not severe, almost imperceptible and 10-is extremely severe, hard to handle.

1……2……3……4…...5……6……..7……8…….9……10

5.9 Did any treatment was applied?

Yes/ No

If 'YES': please provide the name of used medicine;

………………………………………………………………………………………………
………………………………………………………………………………………………
………………………………………………………………………………………………
…………………………..

6. How old was this child/participant/participant when had his/her ***first illness/ health problem*** after eating ***any*** food?

Immediately after birth;
In 1st year of life;
Before 3rd year of life;
3–6th year of life;
Later than 6th year of life
If it is possible precise: …………

6.1 Which of these foods mentioned above in points 3 and 4 was the first causing health problem?

…………………….

6.2 Does this problem was manifested like: (if yes put the symptom in a circle-possible multiple choice)

1- Itching, tingling or swelling in the mouth, lips or throat
2- A rash, nettle sting like rash or itchy skin
3- Diarrhea or vomiting (other than food poisoning)
4- Runny or stuffy nose
5- Red, sore or running eyes
6- Difficulty swallowing
7- Breathlessness
8- Stiffness in his/her joints
9- Fainting or dizziness
10- Headaches
11- Anaphylaxis
12-Other: (if yes please describe) ……………

6.3 How long after eating the food did the first symptom appeared?

-minutes
-hours
-days

6.4 How long did it last?

-minutes
-hours
-days

6.5 How severe the manifestation was:

Use the 10 points scale where 1-is not severe, almost imperceptible and 10-is extremely severe, hard to handle.

1……2……3……4…...5……6……..7……8…….9……10

6.6 Did any treatment was applied?

If yes, please provide the name of used medicine;

…………………………………………………………………………………………………
…………………………………………………………………………………………………
…………………………………………………………………………………………………
……………………………..

7. Does the allergy symptoms worsen after consuming a specific product (not type of food but specific product) –e.g., milk is to general- try to specify- skimmed milk, 2% milk, condensed milk, infant formula milk

…………………………………………………………………………………………………
…………………………………………………………………………………………………
………………

8. Does the allergy symptoms worsen after consuming ***dairy products***?

Yes/ No

If 'YES': go to **8.1** ***If 'NO':*** go to **9.**

8.1 How old was this child/participant/participant when had his/her first illness/ health problem after eating ***dairy products***?

-immediately after birth;
-in 1st year of life;
-before 3rd year of life;
–3–6th year of life;
-later than 6th year of life

If it is possible precise: …………

8.2 Does this problem was manifested like: (if yes put the symptom in a circle-possible multiple choice)

1- Itching, tingling or swelling in the mouth, lips or throat
2- A rash, nettle sting like rash or itchy skin
3- Diarrhea or vomiting (other than food poisoning)
4- Runny or stuffy nose
5- Red, sore or running eyes
6- Difficulty swallowing
7- Breathlessness
8- Stiffness in his/her joints
9- Fainting or dizziness
10- Headaches
11- Anaphylaxis
12-Other: (if yes please describe) ……………

8.3 Which of the following products make the symptoms worse? (possible multiple choice)

1. milk
2. yogurt
3. kefir
4 cottage cheese/ricotta
5. butter
6. buttermilk
7. cheese
8.goat/ sheep or milk of other mammals
9.Other
(what?) ………………………………………………………………………………………

8.4 How long after eating the food did the first symptom appeared?
-minutes
-hours
-days

8.5 How long did it last?
-minutes
-hours
-days

8.6 How severe the manifestation was:
Use the 10 points scale where 1-is not severe, almost imperceptible and 10-is extremely severe, hard to handle.
1……2……3……4…...5……6……..7……8…….9……10

8.7 Did any treatment was applied?
If 'YES' :please provide the name of used medicine;…………………………………………………

9. How much milk / dairy products does the child/participant/participant consume?
- without limits,
- less than a glass a day (less due to ailments)
- less than a glass a day (despite the lack of ailments)
- does not consume at all because of ailments
- does not consume at all despite the lack of ailments

10. How the food allergy was diagnosed first time?
- medical interview
- skin test
- blood serum test
- provocation

- other (what?)

ASTHMA AND ATOPY

11. Has this child/participant/participant ever had wheezing or whistling in his/her chest?

Yes/ No

If 'YES':

11.1 How old was this child/participant/participant when he/she had his/her first attack of wheezing or whistling?

………………….years

11.2 Did any treatment was applied?

If 'YES': please provide the name of used medicine;…………………………………………………

12. Has this child/participant ever had asthma?

Yes/ No

If 'YES':

12.1 How old was this child/participant when he/she had his/her first attack of asthma?

………………….years

12.2 Did any treatment was applied?

If 'YES': please provide the name of used medicine;…………………………………………………

13. Does this child/participant have any nasal allergies?

Yes/ No

If 'YES':

13.1 How old was this child/participant when he/she first had nasal allergy?

………………….years

13.2 Did any treatment was applied?

Yes/ No

If 'YES': please provide the name of used medicine;…………………………………………………

13.3 Has this nasal problem been accompanied by itchy or watery eyes?

Yes/ No

14. Has this child/participant ever had an itchy rash?

Yes/ No

If 'YES':

14.1 How old was this child/participant when he/she first had itchy rash?

………………….years

14.2 Has this child/participant had an itchy rash at any time that affected any of the following places: (possible multiple choice)

- the folds of the elbows
- behind the knees
- in front of the ankles
- around the neck
- around the ears
- around the eyes

15. Has this child/participant ever had eczema?

Yes/ No
If 'YES':
15.1 How old was this child/participant when he/she first had itchy rash**?**
…………………years

16. Does this child/participant have an aggravation of health status in the presence of cat/dog/horse:
Yes/ No
If 'YES':

16.1. What is a manifestation: (possible multiple choice)
- start to cough?
- start to wheeze?
- get a feeling of tightness in his/her chest?
- start to feel short of breath?
- get a runny or stuffy nose or start to sneeze?
- get itchy or watering eyes?

17. Does this child/participant have an aggravation of health status in the presence of pollen:
Yes/ No
If 'YES':

17.1. What is a manifestation: (possible multiple choice)
- start to cough?
- start to wheeze?
- get a feeling of tightness in his/her chest?
- start to feel short of breath?
- get a runny or stuffy nose or start to sneeze?
- get itchy or watering eyes?

18. Has this child/participant ever had immunotherapy for allergy?
What allergens was he/she desensitized for?
- pollen counts
- mites
- latex
- insect venom
- others

19. Over time, has your child/participant experienced any changes in the intensity of the allergy manifestations?
-yes - intensification
-yes - mute
-no
-I do not know

PREGNANCY, BIRTH AND INFANCY

20. How old was the mother when she gave birth to this child/participant?
…………………years

21. In what week of pregnancy the child/participant was born?
…………………week

22. What was the method of birth
-naturally
-by caesarean section

23. How many points on the APGAR scale did child/participant receive after delivery?
………points

24. Was this child/participant ever given **antibiotics** in the first two years of life?
Yes/ No
25. How often the **antibiotic** therapy was implemented:
1 – 2 times a year
-less often than once a year
3 – 4 times a year
-more than 4 times a year

26. Was this child/participant ever given **probiotics** in the first two years of life?
Yes/ No

27. How often **probiotics** were implemented:
1 – 2 times a year
-less often than once a year
3 – 4 times a year
-more than 4 times a year
- daily prophylaxis

28. During the infancy, the child/participant was:
- Only breastfed (if yes go to 27)
- Infant formula fed (if yes go to 28)
- Mixed fed (if yes go to 27 and 28)

29. How long the child/participant was fed naturally?
0 – 2 months
3 – 6 months
-longer than 6 months
30. Which of the following types of infant formula has this child/participant received?
- Normal cow's milk formula
- Normal soy milk formula
- Hypo-allergenic (modified) formula
31. At what age were solid foods provided to the diet?
……………..months
32. Did the mother during pregnancy was taking probiotics?
Yes/No
33. What type of probiotics during pregnancy was taken:
-support for the microbiota of the genital tract
-support for the intestinal microbiota
34. Was the mother during pregnancy smoking?
Yes/No
35. Did mother follow any special diet during pregnancy?
Yes/No
If 'YES' put the diet in a circle-possible multiple choice)
- Weight reducing (low calories)
- Low cholesterol
- Elimination (caused by allergy/intolerance)
- Low sodium
- Diabetic
- High potassium
- Ulcer
- High protein
- High fat
- Vegan

- Vegetarian
- Mediterranean
- Oriental
- Low processed
-Other : ………………………

36. Was it driven by:
- family burden with allergic diseases
- protective effect of the fetus
- protective effect of the pregnant woman
- better well-being of the pregnant woman

HOUSHOLD AND A FAMILY HISTORY OF ATOPY

37. Place of residence of child/participant
-village
-city with 50 -100 thousand residents
-a city with over 100,000 residents

38. Parental education
- Primary school
- Technical
- Secondary
- Postgraduate

39. Professional status of child/participant/participant
-pupil
-student
-working person (manual worker)
-working person (white-collar worker)
-non-working person
-retired pensioner

40. How many brothers and sisters does this child/participant have or did have? Correct answer put in a circle-and add the exact number of siblings)
- only child
- older siblings: ……………..
- younger siblings:…………….
- same age:………………

41. Did this child/participant go to a school, play-school or nursery?
Yes/No

42. Did the child/participant had contact with pets?
Yes/No
If 'YES' please precise what it was:……………………..

43. Did people related to child/participants had ever **food allergies**?
Yes/No
If 'YES'
43.1 Who was that for a child/participant?
-siblings
-mother
-father
-grandparents
-other family member: ……………………..(please specify the degree of relationship)

44. Did people related to child/participants had ever **cows' milk protein allergy**?
Yes/No
If 'YES'
38.1 Who was that for a child/participant?
-siblings
-mother

-father
-grandparents
-other family member: ……………………..(please specify the degree of relationship)

45. Did people related to child/participant had ever **other allergies**?
Yes/No
If 'YES'

45.1 Who was that for a child/participant?
-siblings
-mother
-father
-grandparents
-other family member: ……………………..(please specify the degree of relationship)

46. Does the child/participant suffer any other diseases?
Yes/No
If 'YES'(possible multiple choice)
- circulatory system (ischemic heart disease, arrhythmias, defects
- heart, high blood pressure, heart failure, shortness of breath)
- respiratory system (pulmonary cystic fibrosis, chronic inflammation
- bronchitis, emphysema, sleep apnea)
- nervous and muscular systems (autism, epilepsy, muscular dystrophy)
- digestive system (peptic ulcer disease, inflammatory bowel diseases, malabsorption syndrome)
- cardiovascular system (hemophilia A or B, thrombocytopenia, vascular diathesis, anemia, leukemia, lymphoma)
- the urinary system (renal failure, inflammation of the kidneys)
- infectious diseases (Cytomegalic, hepatitis B or C, tuberculosis, AIDS)
- parasitic diseases
- cancers
- metabolic and endocrine diseases (diabetes, hyperthyroidism,hypothyroidism, hyperlipidemia, obesity, cachexia, adrenal gland disease, pheochromocytoma)
- other:……………………

47. Does the mother of child/participants suffer any other diseases?
Yes/No
If 'YES'(possible multiple choice)
- circulatory system (ischemic heart disease, arrhythmias, defects
- heart, high blood pressure, heart failure, shortness of breath)
- respiratory system (pulmonary cystic fibrosis, chronic inflammation
- bronchitis, emphysema, sleep apnea)
- nervous and muscular systems (autism, epilepsy, muscular dystrophy)
- digestive system (peptic ulcer disease, inflammatory bowel diseases, malabsorption syndrome)
- cardiovascular system (hemophilia A or B, thrombocytopenia, vascular diathesis, anemia, leukemia, lymphoma)
- the urinary system (renal failure, inflammation of the kidneys)
- infectious diseases (Cytomegalic, hepatitis B or C, tuberculosis, AIDS)
- parasitic diseases
- cancers
- metabolic and endocrine diseases (diabetes, hyperthyroidism,hypothyroidism, hyperlipidemia, obesity, cachexia, adrenal gland disease, pheochromocytoma)
- other:……………………

48. Does the child/participant follow any diet?
Yes/No

If 'YES' Please specify what type and go to **48.1**:…………………………………………………

If 'NO' go to **49.**

48.1 When this diet was introduced:……………………………………………………

48.2 What was the reason of diet introduction:………………………………………….

49. Does the child/participant eat meals in restaurant mass catering?

Yes/No

50. Is the child/participant familiar with the term of a 'hidden allergen'?

Yes/No

BIOMETRIC DATA (inclusion/follow up)-completed by clinicians

51. Weight of child/participant

…………….kg

52. Height of child/participant

…………….cm

53. Calculated BMI z-scores

…………………………

54. SCORAD scale if answers in questions **5.5**, **6.2** or **8.2** includes answers (1–2) or ***If 'YES'*** in **14,15** question.

……………………

Appendix A2- FFQ Survey

1. For each food listed, fill in the cross indicating how often on average you have used the amount specified food item during pregnancy.

Table S2 FFQ Survey

		Frequency								
**Food Item**	**Serving size**	**Never or less than once per month**	**1–3 per month**	**1 per week**	**2–4 per week**	**5–6 per week**	**1 per day**	**2–3 per day**	**4–5 per day**	**6+ per day**
Dairy foods										
Skim, low-fat milk	8 oz. glass									
1–2%fat milk	8 oz. glass									
Whole milk	8 oz. glass									
Soy/plant milk	8 oz. glass									
Cream	1 tbs									
Sour cream	1 tbs									
Nondairy coffee whitener	1 tbs									
Sherbet, ice milk	1 cup									
Ice cream	1 cup									
Yogurt plain or low-carb	1 cup									
Yogurt sweetened with fruit	1 cup									
Kefir	1 cup									
Buttermilk	1 cup or 8 oz. glass									
Cottage or ricotta cheese	½ cup									
Cream ceese	1 oz.									
Other cheese like rippened	1 oz. or 1 slice									
Tofu	1 oz. or 1 slice									
Margarine	1 pat									
Butter	1 pat									
Fruits (fresh and dried)										

Apples, pear	1 fruit
Apple juice, cider	Small glass
Apricots	½ fruit
Avocado	1 fruit
Bananas	1 fruit
Blueberries	½ cup
Oranges	1 fruit
Orange juice	Small glass
Grapefruit	½ fruit
Grapefruit juice	Small glass
Grapes, cherries	½ cup
Other fruit juices	Small glass
Strawberries	½ cup
Peaches	1 fruit
Plums	½ cup
Dried raisins	1 oz. or a small pack
Dried plums	1 oz. or a small pack
Vegetables	
Beans	½ cup
Broccoli	½ cup
Brussel sprouts	½ cup
Cabbage (including cooked)	½ cup
Carrots	1 whole, ½ cup
Carrot juice	Small glass
Corn	1 ear, ½ cup
Cauliflower	½ cup
Celery	2–3 stick
Cucumber fresh	3 slices
Eggplant, zucchini, summer squash	½ cup
Garlic	1 clove, 1 shake
Lettuce	1 cup

Onion	½ cup
Peas,	½ cup
Mixed vegetables	½ cup
Sprouts	½ cup
Mushrooms	1 each
Pepeer	3 slices
Yams, sweet potatoes	½ cup cooked
Spinach	½ cup cooked
Kale, mustard, chard greens	½ cup cooked
Tomatoes	1 fruit
Tomato juice	Small glass
Tofu or soybeans	3–4 oz.
Sauerkraut	½ cup
Pickled cucumbers	½ cup
Eggs, meats, fish	
Eggs	1
Chicken, turkey with skin	4–6 oz.
Chicken, turkey without skin	4–6 oz.
Processed meats	1 slice
Bacon	2 slice
Sausages	2
Hamburger	1 patty
Beef, lamb	4–6 oz.
Pork	4–6 oz.
Canned tuna fish	3–4 oz.
Dark meat fish e.g., tuna, mackerel, salmon	3–5 oz.
Other fish e.g., cod, halibut	3–5 oz.

Shrimp, lobster, scallops	3–5 oz.
Breads, cereals, starches	
Cold breakfast cereal	1 cup
Cooked oatmeal	1 cup
Other cooked breakfast cereal	1 cup
White bread including pita	1 slice
Dark bread whole grain	1 slice
Rye/ Pumpernickel bread	1 slice
English muffins, bagels, rolls	1
Muffins, biscuits	1
Pancakes/ waffles	2 small pieces
Tortillas	2
Brown rice	1 cup
White rice	1 cup
Pasta	1 cup
Other grains	1 cup
French fries	6 oz. or one serving
Potatoes (baked, boiled, mashed)	1 whole, 1 cup
Potatoe/ corn Chips	1 small bag
Crackers, pretzels	4
Pizza	2 slices
Beverages	

Low-calorie drinks with caffeine	1 can
Other low-calorie carbonated beverage	1 can
Carbonated drinks with caffeine with sugar	1 can
Carbonated beverage with sugar	1 can
Noncarbonated fruit drinks	1 can
Coffee	1 cup
Black tea not herbal	1 cup
Green tea	1 cup
Herbal tea decaffeinated coffee, cereal coffee	1 cup
Alcoholic drinks	4 oz. glass
Plain water	8 oz. cup
Sweets, baked goods and miscellaneous	
Chocolate	1 oz.
Candy bars	1
Candy, without chocolate	1 oz.
Cookies	1
Pies (homemeade or readymade)	1 slice
Doughnuts	1
Jams, jellies	1 tbs

Honey,	1 tbs
Peanut butter	1 tbs
Peanuts	1 oz.
Walnuts	1 oz.
Other nutrs	1 oz.
Breakfast and energy bars e.g., granola, protein bar	1
Ketchup or chili sauce	1 tbs
Regular mayonnaise	1 tbs
Fat-free mayonnaise	1 tbs
Olive oil	1 tbs
Other vegetable oil	1 tbs
Sugar	1 tbs
Artificial sweetener	1

2. How many teaspoons of sugar did you use to your beverages or food every day

…………….Tbs

3. What type of margarine or spread did you used?
   - Regular
   - Light
   - Nonfat

4. What kind of fat for baking was used at home?
   - Butter
   - Margarine
   -Olive oil
   -Vegetable oil
   -Lard

5. Are there any important foods that you have been eating regularly apart from those mentioned above e.g., horseradish, mango, wheat germ, seaweed, algae, olives …

If yes how often:

Food: Frequency:

………………………………………………… …………………………………………………

6. Did you take multi-vitamins?
   -yes (if yes how many pills per week) ………………………… pills/week
   -no

7. Not counting multi-vitamins did you take any of following preparations (if yes what was the dose)

- Vitamin A – …………… dose/day
- Vitamin B6– …………… dose/day

- Vitamin B9 – …………… dose/day
- B complex – …………..dose/day
- Vitamin C – …………… dose/day
- Vitamin D – …………… dose/day
- Vitamin E– …………… dose/day
- Potassium – …………… dose/day
- Calcium – …………… dose/day
- Zinc – …………… dose/day
- Selenium – …………… dose/day
- Magnesium – …………… dose/day
- Fish oil -……………dose/day
- Flax seed– …………… dose/day
- Yeast flakes– …………… dose/day
-Probiotics– …………… dose/day
-Prebiotics e.g., inulins and FOS ……. dose day
8. Did you follow any special diet during pregnancy?
-Yes/-No If yes which one:
-Weight reducing (low calories)
-Low cholesterol
- Elimination (caused by allergy/intolerance)
-Low sodium
-Diabetic
-High potassium
-Ulcer
-Other : ………………………9. How strictly the special diet was adhered to?
-without derogations
- derogations 1–2 times a week
- derogations 3–4 times a week
- derogations more often than 4 times a week
10. Did you follow any other than standard diet during pregnancy
-Yes/-No
If yes which one:
-high protein
-high fat
-vegan
-vegetarian
- Mediterranean
- Oriental
-low processed
-other: ……………………………
11. How strictly the diet was adhered to?
-without derogations
- derogations 1–2 times a week
- derogations 3–4 times a week
- derogations more often than 4 times a week
12. Was it caused by any medical reason
-Yes/ -No
If yes which one:
- family burden with allergic diseases
-protective effect of the fetus
- protective effect of the pregnant woman
- better well-being of the pregnant woman

Appendix A3- FFQ Survey results

Variables	Units	Compared groups					*p* value*	Observed correlationes	
		Control	SFA	PFA	MFA	Mean serving		Mean serving (frequency) vs. Total IgE**	Mean serving (frequency) vs. Severity of allergy **
**Dairy foods**	**Serving size**	**Number of servings per day**							
Skim, low-fat milk	8 oz. glass	0.37 ± 0.17	0.39 ± 0.18	0.37 ± 0.17	0.39 ± 0.28	0.38 ± 0.17	0.916	0.197	**0.435**
1–2%fat milk	8 oz. glass	0.56 ± 0.54	0.57 ± 0.55	0.57 ± 0.55	0.54 ± 0.52	0.56 ± 0.54	0.795	**0.428**	0.176
Whole milk	8 oz. glass	0.55 ± 0.52	0.55 ± 0.52	0.52 ± 0.49	0.53 ± 0.50	0.54 ± 0.51	0.834	0.254	0.081
Soy/plant milk	8 oz. glass	0.07 ± 0.08	0.07 ± 0.08	0.07 ± 0.08	0.07 ± 0.07	0.07 ± 0.08	0.845	**0.449**	0.453
Cream	1 tbs	0.08 ± 0.01	0.08 ± 0.01	0.08 ± 0.01	0.08 ± 0.01	0.08 ± 0.01	0.946	0.151	0.223
Sour cream	1 tbs	0.16 ± 0.16	0.16 ± 0.16	0.16 ± 0.16	0.17 ± 0.17	0.16 ± 0.16	0.742	0.075	
Nondairy coffee wihitener	1 tbs	0.19 ± 0.22	0.18 ± 0.21	0.19 ± 0.22	0.19 ± 0.22	0.19 ± 0.22	0.805	0.064	
Sherbet, ice milk	1 cup	0.29 ± 0.26	0.29 ± 0.26	0.28 ± 0.25	0.29 ± 0.26	0.29 ± 0.26	0.969	0.185	0.379
Ice cream	1 cup	0.18 ± 0.17	0.18 ± 0.17	0.18 ± 0.17	0.19 ± 0.18	0.18 ± 0.17	0.725	0.215	0.354
Yogurt plain or low-carb	1 cup	0.14 ± 0.14	0.14 ± 0.14	0.14 ± 0.14	0.14 ± 0.14	0.14 ± 0.14	0.929	0.072	0.032
Yogurt sweetened with fruit	1 cup	0.42 ± 0.41	0.41 ± 0.40	0.42 ± 0.41	0.39 ± 0.38	0.41 ± 0.40	0.802	0.209	0.419
Kefir	1 cup	0.26 ± 0.09	0.26 ± 0.09	0.26 ± 0.09	0.26 ± 0.09	0.26 ± 0.09	0.784	0.156	0.056
Buttermilk	1 cup or	0.15 ± 0.09	0.14 ± 0.09	0.16 ± 0.09	0.16 ± 0.10	0.15 ± 0.09	0.373	0.074	0.082
Cottage or ricotta cheese	8 oz. glass	0.21 ± 0.21	0.22 ± 0.22	0.20 ± 0.20	0.20 ± 0.20	0.21 ± 0.21	0.574	0.285	0.185
Cream cheese	½ cup	0.12 ± 0.08	0.11 ± 0.08	0.12 ± 0.08	0.11 ± 0.08	0.12 ± 0.08	0.812	0.159	0.102
Other cheese like rippened	1 oz. or 1 slice	0.44 ± 0.44	0.43 ± 0.43	0.43 ± 0.43	0.44 ± 0.44	0.43 ± 0.43	0.910	0.270	0.112
Tofu	1 oz. or 1 slice	0.09 ± 0.10	0.09 ± 0.10	0.10 ± 0.11	0.09 ± 0.10	0.09 ± 0.10	0.857	**0.380**	0.303
Margarine	1 pat	0.10 ± 0.08	0.09 ± 0.08	0.10 ± 0.08	0.10 ± 0.08	0.10 ± 0.08	0.740	0.217	0.279
Butter	1 pat	0.50 ± 0.68	0.51 ± 0.69	0.48 ± 0.66	0.50 ± 0.68	0.50 ± 0.68	0.942	0.212	0.395
**Fruits (fresh and dried)**									0.083
Apples, pear	1 fruit	0.48 ± 0.71	0.46 ± 0.68	0.50 ± 0.74	0.51 ± 0.75	0.41 ± 0.72	**0.049**	**0.407**	0.175
Apple juice, cider	Small glass	0.29 ± 0.39	0.28 ± 0.38	0.29 ± 0.40	0.27 ± 0.37	0.28 ± 0.38	0.897	0.238	0.374
Apricots	½ fruit	0.13 ± 0.03	0.12 ± 0.03	0.14 ± 0.03	0.12 ± 0.03	0.13 ± 0.03	0.827	0.099	0.263
Avocado	1 fruit	0.23 ± 0.2	0.22 ± 0.19	0.22 ± 0.19	0.24 ± 0.21	0.23 ± 0.20	0.578	0.289	0.153
Bananas	1 fruit	0.43 ± 0.42	0.43 ± 0.42	0.41 ± 0.40	0.43 ± 0.42	0.42 ± 0.41	0.978	0.327	0.121
Blueberries	½ cup	0.09 ± 0.10	0.09 ± 0.10	0.09 ± 0.10	0.09 ± 0.10	0.09 ± 0.10	0.834	0.195	0.204
Oranges	1 fruit	0.4 ± 0.28	0.42 ± 0.29	0.42 ± 0.29	0.40 ± 0.28	0.41 ± 0.29	0.783	**0.378**	0.233
Orange juice	Small glass	0.44 ± 0.34	0.42 ± 0.33	0.42 ± 0.32	0.46 ± 0.35	0.43 ± 0.34	0.586	0.326	0.328
Grapefruit	½ fruit	0.33 ± 0.14	0.35 ± 0.15	0.34 ± 0.14	0.35 ± 0.15	0.34 ± 0.14	0.956	0.036	0.305
Grapefruit juice	Small glass	0.14 ± 0.13	0.13 ± 0.12	0.15 ± 0.14	0.14 ± 0.13	0.14 ± 0.13	0.752	0.063	0.379
Grapes, cherries	½ cup	0.16 ± 0.16	0.17 ± 0.17	0.15 ± 0.15	0.17 ± 0.17	0.16 ± 0.16	0.999	0.054	0.257

Other fruit juices	Small glass	0.03 ± 0.03	0.03 ± 0.03	0.01 ± 0.03	0.03 ± 0.03	0.06 ± 0.09	0.985	0.255	0.399
Strawberries	½ cup	0.15 ± 0.15	0.16 ± 0.16	0.15 ± 0.15	0.15 ± 0.15	0.15 ± 0.15	0.807	0.122	0.182
Peaches	1 fruit	0.12 ± 0.13	0.13 ± 0.14	0.12 ± 0.13	0.11 ± 0.12	0.12 ± 0.13	0.474	0.277	0.133
Plums	½ cup	0.18 ± 0.09	0.17 ± 0.08	0.17 ± 0.09	0.29 ± 0.19	0.18 ± 0.09	0.196	0.289	0.357
Dried raisins	1 oz. or a small pack	0.05 ± 0.05	0.05 ± 0.05	0.05 ± 0.05	0.05 ± 0.05	0.05 ± 0.05	0.905	0.239	0.161
Dried plums	1 oz. or a small pack	0.07 ± 0.08	0.07 ± 0.08	0.07 ± 0.08	0.07 ± 0.08	0.07 ± 0.08	0.989	0.139	0.065
**Vegetables**									0.143
Beans	½ cup	0.11 ± 0.12	0.14 ± 0.12	0.10 ± 0.11	0.15 ± 0.13	0.11 ± 0.12	0.173	**0.375**	0.352
Broccoli	½ cup	0.36 ± 0.42	0.38 ± 0.44	0.38 ± 0.45	0.36 ± 0.42	0.37 ± 0.43	0.848	0.260	0.344
Brussel sprouts	½ cup	0.06 ± 0.16	0.06 ± 0.16	0.06 ± 0.16	0.06 ± 0.16	0.06 ± 0.16	0.991	0.088	0.440
Cabbage (including cooked)	½ cup	0.10 ± 0.15	0.10 ± 0.15	0.10 ± 0.15	0.14 ± 0.15	0.10 ± 0.15	0.911	0.202	0.126
Carrots	1 whole, ½ cup	0.39 ± 0.29	0.41 ± 0.30	0.38 ± 0.28	0.42 ± 0.31	0.40 ± 0.30	0.806	0.292	0.087
Carrot juice	Small glass	0.29 ± 0.14	0.28 ± 0.14	0.34 ± 0.14	0.27 ± 0.13	0.28 ± 0.14	0.270	**0.350**	0.080
Corn	1 ear, ½ cup	0.12 ± 0.09	0.11 ± 0.08	0.11 ± 0.08	0.15 ± 0.10	0.12 ± 0.09	0.320	0.204	0.119
Cauliflower	½ cup	0.19 ± 0.17	0.19 ± 0.17	0.20 ± 0.18	0.18 ± 0.16	0.19 ± 0.17	0.699	0.132	0.277
Celery	2–3 stick	0.14 ± 0.12	0.14 ± 0.12	0.17 ± 0.13	0.15 ± 0.12	0.14 ± 0.12	0.632	**0.392**	0.385
Cucumber fresh	3 slices	0.21 ± 0.11	0.22 ± 0.11	0.20 ± 0.11	0.22 ± 0.12	0.21 ± 0.11	0.961	0.138	0.135
Eggplant, zucchini, summer squash	½ cup	0.09 ± 0.07	0.09 ± 0.07	0.09 ± 0.07	0.09 ± 0.07	0.09 ± 0.07	0.856	0.031	0.192
Garlic	1 clove, 1 shake	0.10 ± 0.11	0.10 ± 0.11	0.10 ± 0.11	0.10 ± 0.11	0.10 ± 0.11	0.839	0.162	0.441
Lettuce	1 cup	0.44 ± 0.43	0.46 ± 0.45	0.47 ± 0.46	0.43 ± 0.42	0.45 ± 0.44	0.695	0.054	0.353
Onion	½ cup	0.27 ± 0.06	0.17 ± 0.06	0.17 ± 0.06	0.18 ± 0.06	0.17 ± 0.06	0.580	0.167	0.098
Mixed vegetables	½ cup	0.25 ± 0.29	0.24 ± 0.28	0.25 ± 0.29	0.26 ± 0.30	0.25 ± 0.29	0.772	0.021	0.156
Sprouts	½ cup	0.09 ± 0.40	0.09 ± 0.41	0.09 ± 0.40	0.10 ± 0.43	0.09 ± 0.41	0.926	0.188	0.121
Mushrooms	½ cup	0.10 ± 0.09	0.11 ± 0.09	0.10 ± 0.09	0.10 ± 0.09	0.10 ± 0.09	0.763	0.170	0.196
Pepeer	1 each	0.14 ± 0.14	0.14 ± 0.14	0.14 ± 0.14	0.15 ± 0.15	0.14 ± 0.14	0.741	0.425	0.322
Yams, sweet potatoes	3 slices	0.12 ± 0.12	0.12 ± 0.12	0.11 ± 0.11	0.13 ± 0.13	0.12 ± 0.12	0.754	0.162	0.303
Spinach	½ cup cooked	0.11 ± 0.21	0.11 ± 0.20	0.12 ± 0.22	0.11 ± 0.22	0.11 ± 0.21	0.906	0.210	0.222
Kale, mustard, chard greens	½ cup cooked	0.03 ± 0.03	0.03 ± 0.03	0.03 ± 0.03	0.03 ± 0.03	0.03 ± 0.03	0.881	0.306	0.091
Tomatoes	½ cup cooked	0.47 ± 0.26	0.45 ± 0.25	0.46 ± 0.25	0.45 ± 0.25	0.46 ± 0.25	0.923	0.078	0.332
Tomato juice	1 fruit	0.17 ± 0.07	0.18 ± 0.07	0.17 ± 0.07	0.18 ± 0.07	0.18 ± 0.07	0.966	0.064	0.200
Tofu or soybeans	Small glass	0.08 ± 0.18	0.08 ± 0.19	0.07 ± 0.17	0.08 ± 0.18	0.08 ± 0.18	0.892	**0.366**	0.297
Sauerkraut and cucumbers	3–4 oz.	0.20 ± 0.10	0.21 ± 0.10	0.23 ± 0.10	0.19 ± 0.09	0.20 ± 0.10	0.305	0.070	0.216
Pickled cucumbers	½ cup	0.32 ± 0.11	0.31 ± 0.11	0.34 ± 0.12	0.36 ± 0.10	0.32 ± 0.11	0.060	0.203	0.152
**Eggs, meats, fish**									
Eggs	1	0.29 ± 0.32	0.28 ± 0.31	0.30 ± 0.34	0.29 ± 0.32	0.29 ± 0.32	0.864	0.019	0.267
Chicken, turkey with skin	4–6 oz.	0.21 ± 0.19	0.21 ± 0.19	0.21 ± 0.19	0.21 ± 0.19	0.21 ± 0.19	0.916	0.247	0.166
Chicken, turkey without skin	4–6 oz.	0.25 ± 0.16	0.27 ± 0.17	0.26 ± 0.16	0.26 ± 0.17	0.26 ± 0.17	0.831	0.104	0.247
Processed meats	1 slice	0.16 ± 0.06	0.15 ± 0.06	0.17 ± 0.06	0.17 ± 0.06	0.16 ± 0.06	0.169	0.042	**0.350**
Bacon	2 slice	0.10 ± 0.12	0.09 ± 0.11	0.10 ± 0.12	0.10 ± 0.12	0.10 ± 0.12	0.819	0.214	0.207
Sausages	2	0.15 ± 0.16	0.15 ± 0.16	0.15 ± 0.15	0.14 ± 0.15	0.15 ± 0.16	0.719	0.285	0.152

Hamburger	1 patty	0.21 ± 0.12	0.20 ± 0.12	0.22 ± 0.13	0.20 ± 0.11	0.21 ± 0.12	0.883	0.076	0.223
Beef, lamb	4–6 oz.	0.39 ± 0.41	0.39 ± 0.41	0.38 ± 0.39	0.39 ± 0.41	0.39 ± 0.41	0.970	0.160	0.268
Pork	4–6 oz.	0.11 ± 0.11	0.11 ± 0.11	0.11 ± 0.11	0.11 ± 0.11	0.11 ± 0.11	0.855	0.104	0.122
Canned tuna fish	3–4 oz.	0.25 ± 0.21	0.26 ± 0.22	0.25 ± 0.21	0.24 ± 0.20	0.25 ± 0.21	0.614	0.244	0.102
Dark meat fish e.g., tuna, mackerel, salmon	3–5 oz.	0.15 ± 0.15	0.14 ± 0.14	0.20 ± 0.16	0.15 ± 0.15	0.15 ± 0.15	0.784	-0.211	0.232
Other fish e.g., cod, halibut	3–5 oz.	0.16 ± 0.16	0.26 ± 0.16	0.15 ± 0.15	0.15 ± 0.15	0.16 ± 0.16	0.648	0.006	0.151
Shrimp, lobster,scallops	3–5 oz.	0.04 ± 0.14	0.04 ± 0.14	0.04 ± 0.14	0.04 ± 0.13	0.04 ± 0.14	0.988	0.127	0.155
**Breads, cereals, starches**									
Cold breakfast cereal	1 cup	0.37 ± 0.38	0.34 ± 0.35	0.36 ± 0.37	0.38 ± 0.39	0.36 ± 0.37	0.629	0.273	0.268
Cooked oatmeal	1 cup	0.18 ± 0.18	0.17 ± 0.17	0.19 ± 0.19	0.17 ± 0.17	0.18 ± 0.18	0.924	0.342	0.108
Other cooked breakfast cereal	1 cup	0.09 ± 0.09	0.09 ± 0.09	0.09 ± 0.09	0.09 ± 0.09	0.09 ± 0.09	0.949	0.197	0.074
White bread including pita	1 slice	0.71 ± 0.31	0.74 ± 0.32	0.74 ± 0.32	0.74 ± 0.32	0.73 ± 0.32	0.952	**0.415**	0.356
Dark bread whole grain	1 slice	0.80 ± 0.29	0.85 ± 0.31	0.79 ± 0.29	0.81 ± 0.29	0.81 ± 0.29	0.461	0.098	0.226
Rye/ Pumpernickel bread	1 slice	0.08 ± 0.09	0.08 ± 0.09	0.08 ± 0.09	0.08 ± 0.09	0.08 ± 0.09	0.913	0.138	0.177
English muffins, bagels, rolls	1	0.18 ± 0.13	0.17 ± 0.12	0.17 ± 0.13	0.18 ± 0.13	0.18 ± 0.13	0.759	0.088	0.340
Muffins, biscuits	1	0.27 ± 0.26	0.27 ± 0.26	0.29 ± 0.28	0.27 ± 0.26	0.27 ± 0.26	0.991	0.171	0.131
Pancakes/ waffles	2 small pieces	0.64 ± 0.69	0.65 ± 0.71	0.63 ± 0.68	0.67 ± 0.72	0.65 ± 0.70	0.909	0.214	0.461
Tortillas	2	0.08 ± 0.08	0.07 ± 0.07	0.08 ± 0.08	0.08 ± 0.08	0.08 ± 0.08	0.734	0.184	0.197
Brown rice	1 cup	0.12 ± 0.09	0.12 ± 0.09	0.13 ± 0.09	0.11 ± 0.08	0.12 ± 0.09	0.643	0.173	0.418
White rice	1 cup	0.19 ± 0.11	0.19 ± 0.11	0.20 ± 0.11	0.20 ± 0.11	0.19 ± 0.11	0.561	0.339	0.262
Pasta	1 cup	0.20 ± 0.19	0.21 ± 0.20	0.20 ± 0.19	0.21 ± 0.20	0.21 ± 0.19	0.969	0.212	0.021
Other grains	1 cup	0.37 ± 0.31	0.39 ± 0.33	0.36 ± 0.30	0.35 ± 0.30	0.37 ± 0.31	0.491	0.292	0.196
French fries	6 oz. or one serving	0.31 ± 0.34	0.32 ± 0.35	0.29 ± 0.32	0.31 ± 0.34	0.31 ± 0.34	0.878	0.199	0.327
Potatoes (baked, boiled, mashed)	1 whole, 1 cup	0.47 ± 0.49	0.49 ± 0.51	0.44 ± 0.46	0.48 ± 0.50	0.47 ± 0.49	0.926	0.283	0.115
Potatoe/ corn Chips	1 small bag	0.39 ± 0.35	0.38 ± 0.34	0.37 ± 0.33	0.39 ± 0.35	0.38 ± 0.34	0.921	0.245	0.186
Crackers, pretzels	4	0.11 ± 0.11	0.10 ± 0.10	0.11 ± 0.11	0.10 ± 0.10	0.11 ± 0.11	0.820	0.127	0.110
Pizza	2 slices	0.22 ± 0.18	0.22 ± 0.18	0.21 ± 0.17	0.21 ± 0.17	0.21 ± 0.18	0.797	0.260	0.340
**Beverages**									
Low-calorie drinks with caffeine	1 can	0.10 ± 0.10	0.10 ± 0.00	0.10 ± 0.00	0.10 ± 0.00	0.10 ± 0.00	0.999	0.142	0.228
Other low-calorie carbonated beverage	1 can	0.16 ± 0.17	0.16 ± 0.17	0.16 ± 0.17	0.16 ± 0.17	0.16 ± 0.17	0.962	0.284	0.251
Carbonated drinks with caffeine with sugar	1 can	0.12 ± 0.12	0.12 ± 0.12	0.11 ± 0.11	0.13 ± 0.13	0.12 ± 0.12	0.836	0.216	0.255
Carbonated beverage with sugar	1 can	0.17 ± 0.17	0.16 ± 0.16	0.17 ± 0.17	0.18 ± 0.18	0.17 ± 0.17	0.528	0.259	0.210
Noncarbonated fruit drinks	1 can	0.27 ± 0.26	0.27 ± 0.26	0.27 ± 0.26	0.28 ± 0.27	0.27 ± 0.26	0.879	0.193	0.227
Coffee	1 cup	0.93 ± 0.79	0.99 ± 0.84	0.94 ± 0.79	0.90 ± 0.76	0.94 ± 0.80	0.545	0.187	0.195
Black tea not herbal	1 cup	1.73 ± 1.51	1.64 ± 1.43	1.70 ± 1.48	1.67 ± 1.46	1.68 ± 1.47	0.913	0.161	0.121
Green tea	1 cup	0.32 ± 0.26	0.28 ± 0.27	0.26 ± 0.25	0.27 ± 0.26	0.27 ± 0.26	0.149	-0.326	0.107
Herbal tea decaffeinated coffee, cereal coffee	1 cup	0.99 ± 1.05	0.93 ± 0.99	1.02 ± 1.08	1.02 ± 1.08	0.99 ± 1.05	0.669	0.183	0.224
Alcoholic drinks	4 oz. glass	0.02 ± 0.03	0.02 ± 0.03	0.02 ± 0.03	0.00 ± 0.03	0.00 ± 0.03	0.924	0.211	0.138
Plain water	8 oz. cup	2.92 ± 2.81	2.85 ± 2.74	3.02 ± 2.91	2.82 ± 2.71	2.90 ± 2.79	0.953	0.119	0.206
**Sweets, baked goods and miscellaneous**									

Chocolate	1 oz.	0.32 ± 0.35	0.31 ± 0.33	0.31 ± 0.33	0.31 ± 0.34	0.31 ± 0.34	0.942	0.267	0.103
Candy bars	1	0.13 ± 0.13	0.14 ± 0.14	0.12 ± 0.12	0.13 ± 0.13	0.13 ± 0.13	0.808	0.280	0.193
Candy, without chocolate	1 oz.	0.38 ± 0.35	0.39 ± 0.36	0.39 ± 0.36	0.38 ± 0.35	0.38 ± 0.35	0.922	0.216	0.263
Cookies	1	0.41 ± 0.38	0.42 ± 0.39	0.38 ± 0.36	0.41 ± 0.38	0.41 ± 0.38	0.897	0.227	0.207
Pies (homemeade or readymade)	1 slice	0.29 ± 0.29	0.29 ± 0.29	0.29 ± 0.29	0.30 ± 0.30	0.29 ± 0.29	0.931	0.266	0.192
Doughnuts	1	0.13 ± 0.12	0.13 ± 0.12	0.14 ± 0.13	0.13 ± 0.12	0.13 ± 0.12	0.887	0.186	0.181
Jams, jellies	1 tbs	0.13 ± 0.14	0.13 ± 0.15	0.12 ± 0.13	0.13 ± 0.14	0.13 ± 0.14	0.861	0.062	0.197
Honey	1 tbs	0.12 ± 0.11	0.12 ± 0.11	0.12 ± 0.11	0.12 ± 0.11	0.12 ± 0.11	0.837	0.028	0.184
Peanut butter	1 tbs	0.14 ± 0.13	0.13 ± 0.12	0.15 ± 0.14	0.14 ± 0.13	0.14 ± 0.13	0.774	0.214	0.218
Peanuts	1 oz.	0.23 ± 0.09	0.13 ± 0.09	0.16 ± 0.09	0.13 ± 0.09	0.13 ± 0.09	0.277	**0.356**	0.308
Walnuts	1 oz.	0.29 ± 0.23	0.23 ± 0.23	0.24 ± 0.24	0.23 ± 0.23	013 ± 0.23	0.160	**0.354**	**0.395**
Other nuts	1 oz.	0.02 ± 0.02	0.02 ± 0.02	0.02 ± 0.02	0.02 ± 0.02	0.02 ± 0.02	0.772	0.017	0.271
Breakfast and energy bars e.g., granola, protein bar	1	0.11 ± 0.11	0.12 ± 0.12	0.10 ± 0.10	0.12 ± 0.12	0.11 ± 0.11	0.905	0.120	0.165
Ketchup or chili sauce	1 tbs	0.34 ± 0.25	0.35 ± 0.26	0.36 ± 0.27	0.33 ± 0.25	0.35 ± 0.25	0.679	0.197	0.289
Regular mayonnaise	1 tbs	0.24 ± 0.25	0.26 ± 0.27	0.23 ± 0.23	0.25 ± 0.26	0.24 ± 0.25	0.910	0.230	0.267
Fat-free mayonnaise	1 tbs	0.05 ± 0.05	0.05 ± 0.05	0.05 ± 0.05	0.05 ± 0.05	0.05 ± 0.05	0.881	0.261	**0.447**
Olive oil	1 tbs	0.35 ± 0.26	0.22 ± 0.24	0.27 ± 0.26	0.27 ± 0.26	0.27 ± 0.26	0.078	-0.317	-0.165
Other vegetable oil	1 tbs	0.31 ± 0.34	0.29 ± 0.32	0.31 ± 0.34	0.32 ± 0.35	0.31 ± 0.34	0.634	0.363	0.074
Sugar	1 tbs	0.22 ± 0.28	0.23 ± 0.29	0.21 ± 0.27	0.21 ± 0.27	0.22 ± 0.28	0.732	0.094	0.229
Artificial sweetener	1	0.09 ± 0.8	0.08 ± 0.75	0.09 ± 0.79	0.09 ± 0.81	0.09 ± 0.79	0.971	0.145	0.338
**Supplements:**								Qualitative [c]	
multi-vitamins	%	48.7	48.9	60.9	55	53.38	0.474	0.293	0.251
Folic acid	%	48.7	53.2	50	46.7	49.65	0.821	0.295	0.266
Vitamin D	%	37.4	31.9	34.7	33.3	34.33	0.751	0.281	0.148
Fish Oil	%	26.6	27.7	32.6	30	29.23	0.876	-0.171	0.080
Probiotics	%	12.7	25.5	34.8	41.7	28.68	**0.002**	**-0.411**	-0.285
**Diets:**									
Elimination diet	%	6.7	10.6	34.8	38.3	22.60	**< 0.001**	**0.361**	**0.596**
Diabetic	%	35.3	36.2	41.3	31.7	36.13	0.812	0.304	**0.441**
Cholestatic (low fat)	%	4.7	6.4	23.9	15	12.50	**0.006**	0.339	0.398
Vega/Vegearian	%	12.7	2.1	8.7	14	9.38	0.175	**0.416**	0.336
Mediterranean	%	3.3	0	2.2	0	1.38	0.9	0.112	0.215
Diet adherence	a.u (neglects/week)	2.4 ± 0.7	2.6 ± 0.5	2.9 ± 0.5	3.8 ± 0.6	4.9 ± 0.6	0.016	0.293	**0.489**

SFA–allergy to single food-origin allergen, PFA–polyallergy to various food-origin allergens, MFA–mixed polyallergy to aero- and food-origin allergens. Data are presented as the means ±SD or percentages. * *p* values for tests [a] of the Chi-Square test for comparison between groups of nominal data. [b] *p* value of the Kruskal- Wallis test for quantitative data for comparison between groups with Bonferroni's corrected significance threshold of 0.008. ** Pearson's *r* results and [c] for qualitative variables Spearman Significant associations are marked in bold.

Appendix A4- Immunological parameters associations

Table S4. Clinical parameters correlation by health status categories.

Pearson Correlation Factors		Control (*n*-150)			Allergy (*n*-153)		
		r	r^2	*p*	r	r^2	*p*
Total IgE ×	IL-2	0.094	0.009	0.255	**0.185**	**0.034**	**0.001**
	IL-4	−0.093	0.009	0.260	**0.439**	**0.193**	**< 0.001**
	IL-8	0.072	0.005	0.400	**0.399**	**0.160**	**< 0.001**
	INF-γ	0.131	0.017	0.110	**0.162**	**0.026**	**0.005**
Total IgG ×	IL-2	**0.237**	**0.056**	**0.003**	−0.115	0.013	0.159
	IL-4	0.051	0.003	0.539	**0.343**	**0.118**	**< 0.001**
	IL-8	−0.043	0.002	0.604	**0.545**	**0.297**	**< 0.001**
	INF-γ	0.062	0.004	0.450	0.077	0.006	0.344
IL-2 ×	IL-4	−0.142	0.020	0.083	0.196	0.038	0.015
	IL-8	−0.099	0.010	0.226	−0.112	0.013	0.168
IL-4 ×	INF-γ	−0.035	0.001	0.670	**0.496**	**0.246**	**< 0.001**
IL-8 ×	INF-γ	−0.057	0.003	0.490	**0.365**	**0.133**	**< 0.001**

Significant associations of Pearson correlation parameters (r, r^2, *p*) with 95% Confidence are marked in **bold**.

Appendix 5- The contribution of variables in not major factors of multivariate analyses

Table S5. Cluster description of multivariate analysis (PCA) with IgG level as a grouping variable in allergy profile associated to gestational and early life dietary behavior.

Cluster	Immunological Parameters	Dieting and other Variables	*r* values that are different from 0 with a significance level alpha = 0.05
**i**	high total IgG but low total IgE, strong reaction (cl. 4–5) to latex and citruses but also to *Aspergillus fumigatus* (cl.3–4), low to moderate content of IFNγ and IL-2	subjects on mixed and infant formula feeding type, revealed overweight BMI status	0.549
**ii**	high total IgG, moderate total IgE, medium (cl. 2- 4) of antibodies to apple, carrot and to *Aspergillus fumigatus* and high(cl.4–5) to birch and, *Cladosporium,* high content of IL-2/IFNγ	subjects on mixed and infant formula feeding type, maternal gestational diabetic and cholestatic diets, subjects revealed overweight BMI status	0.434
**iii**	moderate total IgG, moderate total IgE, low (cl. 1–2) of antibodies to milk and high to peanut and soybean (cl. 4–5), moderate level of cytokines IL-4/IL-8/IFNγ	maternal gestational cholestatic / vegetarian diets and supplemented with probiotics during pregnancy	0.348

**iv**	low total IgG, low total IgE, moderate (cl. 2–3 level of antibodies to tomato, milk and sesame, moderate level of IL4 and IL-8	maternal standard or elimination diet, subjects mostly female	0.373
**v**	low total IgG, low total IgE, but medium (cl. 3–4) level of antibodies to tomato, sesame, milk - BSA and high (cl. 4–5) to dust mites, moderate level of IL4 and IL-8	maternal standard or elimination diet, subjects mostly female	0.414
**vi**	moderate total IgG, high total IgE, high (cl. 3–5) levels of anti-cows' milk proteins, anti-eggs and anti-nuts antibodies but also strong reaction to pollen and dander (cl. 4–6), high level of IL4 and IL-8	maternal gestational diabetic and cholestatic diets, subjects revealed underweight BMI status and supplemented with probiotics	0.626
**vii**	low total IgG, low total IgE, low (cl.1–2) of antibodies to milk and beef, high level of IL4 and IL-8	subjects breastfed, mothers were on vegan/ vegetarian and or eliminating diet and took probiotics	0.482

Appendix 6- The significance of associations in clusters in multivariate analysis with grouping variable IgG

Table S6. 3–9 Factores for PCA analysis presented in Figure 3.

	**F3**	**F4**	**F5**	**F6**	**F7**	**F8**	**F9**
Grass mix (gx)	0.214	0.106	0.451	1.018	0.100	0.161	0.226
Birch (t3)	0.543	0.862	0.667	0.128	0.228	0.088	0.486
Mugwort (w6)	0.293	0.170	0.844	0.169	0.311	3.509	0.005
Dermatophagoides pter. (d1)	2.355	5.689	0.020	4.281	0.014	0.151	0.108
Dermatophagoides farinae (d2)	3.474	5.804	0.161	2.971	0.005	0.294	0.004
Cat (e1)	1.075	3.423	0.084	2.298	0.035	0.000	1.274
Dog (e2)	4.091	0.011	0.078	0.025	0.544	0.051	0.293
Horse (e3)	0.087	0.335	1.479	0.359	0.016	0.326	2.221
Cladosporium herbarum (m2)	0.074	4.100	0.014	0.018	1.067	13.063	0.454
Aspergillus fumigatus (m3)	5.480	0.091	1.335	0.893	0.061	0.295	0.031
Alternaria alternata (m6)	0.618	0.374	2.022	0.000	0.340	0.365	1.056
Latex (u85)	11.241	1.531	0.003	0.450	0.533	2.380	0.085
Egg white (f1)	1.981	13.319	0.019	0.232	0.563	0.006	0.223
Egg yolk (f75)	5.748	7.144	0.001	0.040	2.416	1.754	1.249
Codfish (f3)	2.228	0.202	1.042	1.796	1.147	11.855	0.237
Shrimp/Prawn (f24)	0.363	1.770	0.132	1.792	0.782	3.511	0.056
Beef (f27)	3.856	4.036	1.056	1.800	3.903	0.004	8.730
Cow's mik (f2)	0.006	1.061	3.428	2.142	1.713	0.283	11.824
Alfa-lactalbumin (f76)	2.858	4.272	1.498	5.079	0.329	7.264	4.653

Beta-lactoglobulin (f77)	0.853	5.034	0.142	1.543	5.656	0.092	0.880
Casein (f78)	2.193	0.257	0.420	4.163	3.245	2.178	6.716
BSA (e204)	9.690	0.013	1.352	1.525	0.007	0.348	2.695
Wheat flour (f4)	0.705	2.261	0.346	2.660	5.361	3.846	0.198
Rice (f9)	0.255	2.249	0.267	2.897	3.464	0.256	0.383
Sesame (f10)	1.872	0.010	0.905	0.087	8.734	1.282	1.972
Soybean (f14)	0.004	1.412	6.147	0.954	8.151	2.015	0.608
Peanut (f13)	0.341	0.019	2.969	5.555	4.041	0.001	0.118
Hazelnut (f17)	1.281	2.277	0.043	1.523	0.352	0.150	0.506
Carrot (f31)	4.877	0.025	0.464	1.009	0.254	0.533	0.002
Potato (f35)	0.008	2.195	0.295	1.418	0.606	3.454	1.613
Tomato (f25)	1.350	0.292	3.249	0.239	15.350	1.993	0.063
Apple (f49)	0.131	0.014	1.177	4.701	0.000	0.016	4.214
Kiwi (f84)	10.944	1.101	0.018	0.789	0.564	1.559	0.011
Elimination diet (M)	0.491	4.372	0.417	0.003	1.154	0.337	5.873
Gestational diabetes diet (M)	0.452	0.224	0.120	0.183	0.850	21.015	0.493
Cholestatic diet (M)	0.082	0.944	0.012	3.769	4.073	6.492	0.132
Vegan/vegetarian diet (M)	0.170	1.228	0.690	0.717	4.931	6.086	1.550
Other diet	0.005	0.033	1.527	0.034	0.634	0.001	1.287
Birth method: natural	0.482	0.027	13.506	0.040	2.104	0.628	0.136
Breasrtfeeding	5.741	2.839	17.416	5.750	2.061	0.012	4.947
Mixed feeding	0.944	6.060	0.883	11.270	0.275	0.145	24.014
Formulas	3.166	0.303	14.904	0.403	4.783	0.261	5.752
Elimination diet (S)	6.438	0.469	3.765	3.738	0.061	1.080	1.345
Chronic diseases (M)	0.222	6.094	3.249	8.144	1.498	0.253	0.113
Chronic diseases (*p*)	0.001	4.472	5.303	10.096	4.298	0.386	0.958
Probiotics (M)	0.713	0.579	2.278	0.114	0.812	0.167	0.189
Probiotics (*p*)	0.006	0.899	3.801	1.185	2.575	0.054	0.015
Variability (%)	8.8	8.0	7.7	7.3	7.0	6.7	5.3
Cumulative (%) with added (Figure 1 and Figure 2).	48.4	56.4	64.0	71.4	78.3	85.0	90.3

Appendix A7- The significance of associations in clusters in neural network map with grouping variable IgG

Table S7. Cluster description of multivariate analysis with (ESOM maps) IgG level as a grouping variable in allergy profile associated to gestational and early life dietary behavior.

Cluster	Immunological parameters	Dieting and other variables	*r* values that are different from 0 with a significance level $\alpha = 0.05$
a	low level (cl. 1–2) of specific E antibodies to egg, low to moderate level (cl. 2–4) to potato, moderate (cl. 3–4) to cow's milk and its' particular proteins (α-La, β-Lg, BSA), moderate total IgG (10–20 g/L), high level of IL-2/IFNγ	maternal gestational diabetic diet and used probiotics during pregnancy.	0.465

b	healthy individuals and allergic subjects with low (cl. 1–2) level of antibodies to cow's milk and carrot, moderate (cl. 3–4) to apple, and high (cl. 4–5) to latex and citruses. All subjects (allergic and controls) had high level of total G antibodies (> 20 g/L), medium levels of IL-2/IFNγ	subjects on mixed and infant formula feeding type, revealed overweight BMI status and supplemented with probiotics	0.377
c	low (cl. 1–2) levels of anti β-Lg antibodies, medium (cl. 2- 4) to apple, birch, wheat flour, and high to egg proteins and nuts (cl. 4- 6), high total IgG (> 20 g/L) and IL-2/IFNγ	maternal gestational diabetic and cholestatic diets	0.506
d	low (cl. 1–2) level of antibodies to cat and dog dander, medium (cl. 3–4) to potato, rice, dust mites and to eggs and high to peanut and soybean (cl. 4–5), high level of cytokines IL-4/IL-8/IFNγ and total G antibodies (> 20 g/L)	maternal gestational cholestatic / vegetarian diets and supplemented with probiotics during pregnancy.	0.412
e	moderate (cl. 3–4) level of antibodies to cow's milk and carrot, dust mite and beef, high levels of IL-4/IL-8/IFNγ but moderate total IgG (10–20 g/L)	subjects were mixed and breastfeed, took probiotics, revealed underweight BMI status, and their mothers were on gestational diabetic diet.	0.249
f	healthy individuals and allergic subjects with low (cl. 1–2) level of antibodies to potato, apple, rice, medium (cl. 3–4) to molds and pollen and high (cl. 5–6) to cat and dog dander. All subjects (allergic and controls) had increased levels of IL-2/IL-4/ IL-8/ IFNγ but moderate level of total G antibodies (10–20 g/L)	maternal vegetarian diets during pregnancy	0.356
g	healthy individuals and allergic subjects with medium (cl. 3–4) level of antibodies to molds, pollen and dust mites and high (cl. 4–5) to casein and cow's milk. All subjects characterized low to moderate total G antibodies (< 10 g/L) and cytokines level.	maternal standard or elimination diet	0.390

Appendix A8- The significance of associations in clusters in multivariate analysis with grouping variable IgE

Table S8. Cluster description of multivariate analysis (PCA) IgE level as a grouping variable in allergy profile associated to gestational and early life dietary behavior.

Cluster	Immunological parameters	Dieting and other variables	*r* values that are different from 0 with a significance level α = 0.05
I	low to moderate total IgE and low IgG content and cytokines	subjects on mixed feeding type, supplemented with probiotics, whose mothers followed standard diet	0.306
II	high total IgE, moderate IgG, low (cl. 1–2) antibodies to eggs, moderate (cl.3–4) content of the antibodies to carrot, apple, soybean and high (cl.5–6) to nuts, pollen and dander and dust mites, with high content of cytokines	subjects supplemented with probiotics, high ratio of male cases, whose mothers followed vegan/vegetarian and elimination diet and took probiotics,	0.603
III	high total IgE, moderate total IgG, medium (cl. 2–3) level of antibodies to tomato, sesame, cows' milk proteins and high (cl. 4–5) to sesame, very high levels of IFNγ, IL4 and IL-8	subjects on mixed and infant formula feeding type, following elimination diet, whose mothers followed gestational diabetic diet, subjects revealed overweight BMI status	0.361
IV	high total IgE, moderate total IgG, medium to high content (cl. 3–5) of antibodies to cows' milk, eggs and wheat	subjects mostly female, whose mothers followed gestational diabetic/cholestatic diet	0.293

Appendix A9. The significance of associations in clusters in neural network map with grouping variable IgE.

Table S9. Cluster description of multivariate analysis (ESOM maps) with IgE level as a grouping variable in allergy profile associated to gestational and early life dietary behavior.

Cluster	Immunological parameters	Dieting and other variables	*r* values that are different from 0 with a significance level α = *0.05*
A	low or no specific antibodies in serum, (cl. 1–2) to dust mites, potato, wheat flour, low IgG but high total IgE, IL-4, IL-8 and IFNγ	subjects with allergy symptoms, on elimination diet and using pharmacotherapy, born by caesarean section, infant formula fed, without a positive family history but also whose mothers were on standard diet and took probiotics during pregnancy	0.496

B	healthy individuals with normal antibodies and cytokines levels	subjects with no positive allergy history and symptoms, naturally born, mixed and breastfeed, and whose mothers were on standard diet and took probiotics during pregnancy	0.314
C	low (cl. 1–2) level of antibodies to molds and rice, moderate (cl. 3–4) to nuts, soybean and high (cl. 5–6) to dander and pollen, with moderate IgG (10–20 g/l) and high total IgE and both IL-2 and IL-4	subjects remaining on an elimination diet for various food allergens like nuts, soybean but also milk, pharmacologically treated and indicating the co-occurrence of other diseases *e.g.* diabetes and hypothyroidism without a confirmed family history of allergy, mostly female, naturally born, breastfed and taking probiotics in infancy, whose mothers were on elimination diet and took probiotics	0.501
D	low (cl. 1–2) level of antibodies to tomato, carrot, apple and moderate to high (cl. 3–5) levels of antibodies to grass and birch pollen, mites. They had moderate IgG (10–20 g/l) and high total IgE and both IL-2 and IL-4	subjects on an elimination diet (S) for various food allergens but consuming milk, mostly male, indicated the co-occurrence of other diseases but negative family history of allergy, revealed underweight BMI status. Childbirth was natural and children were breastfed during infancy. Mothers took probiotics during pregnancy and were on gestational diabetic diet	0.289
E	moderate (cl. 3–4) level of antibodies to nuts, soybean, rice, tomato and high (cl. 5–6) to dander, mites and pollen, with moderate IgG (10–20 g/l) and high total IgE and all tested cytokines	subjects pharmacologically treated, with overweight BMI status, born by caesarean section, breastfed and taking probiotics in infancy, whose mothers were on elimination diet and took probiotics	0.315
F	healthy individuals with normal antibodies and cytokines levels	subjects with no positive allergy history and symptoms, naturally born but infant formula fed, whose mothers were on standard diet	0.366
G	low (cl. 1–2) level of antibodies to cow's milk proteins and rice and low IgG antibodies and cytokines levels	subjects with positive family history of allergy, revealed underweight BMI status, staying on elimination diet , pharmacologically treated, born naturally	0.428

		and fed a mixed diet, whose mothers eliminated milk	
H	with no specific IgE antibodies found in serum, with low IgG but high total IgE, IL-4 and IFNγ	subjects without manifestation of allergy, staying on dairy and gluten-free diet. Mostly female subjects, fed with infant formulas and used probiotics and nutrients, whose mothers followed gestational diabetic diet	0.346
I	low (cl. 1–2) level of antibodies to potato, wheat flour, dust mites, with low IgG and moderate IgE, IL-4, IL-8 and IFNγ	subjects with allergy symptoms, on elimination wheat diet or pharmacotherapy, with positive allergic family history, naturally born, breastfeed, whose mothers were on standard diet during pregnancy	0.272
J	low (cl. 1–2) level of antibodies to milk proteins and eggs, moderate (cl. 3) to carrot, apple and high (cl. 4–5) levels of antibodies to birch pollen, dander and nuts, with moderate IgG (10–20 g/l) and high total IgE and both IL-2 and IL-4	subjects on an elimination diet for various food allergens, revealed underweight BMI status, mostly female, indicate the co-occurrence of other diseases but negative family history of allergy. Born by caesarean section and infant formula fed in infancy. The mothers were on gestational diabetic diet	0.297
K	low (cl. 1–2) level of antibodies to milk proteins and tomato, moderate (cl. 3) to beef, soybean, and peanut and high (cl. 4–5) levels of antibodies to grass, birch pollen, molds, eggs and hazelnuts, with low IgG and high total IgE and IL-4	subjects with allergy symptoms, using pharmacotherapy, mostly male, without a positive family history, naturally born, breastfeed, suffering from other chronic diseases but also whose mothers were on vegan/vegetarian diet during pregnancy	0.215
L	low (cl. 1–2) level of antibodies to tomato and molds, moderate (cl. 3) to beef, soybean, and peanut and high (cl. 4–5) levels of antibodies to grass, birch pollen, molds and hazelnuts, with low IgG but high total IgE, IL-4, IL-8 and IFNγ	subjects with allergy symptoms, on an elimination diet for various food allergens, mostly female, without a positive family history, naturally born, breastfeed, suffering from other chronic diseases but also whose mothers were on vegan/vegetarian diet and took probiotics during pregnancy	0.291

M	low (cl. 1–2) level of antibodies to dust mites, BSA, beef and potato, high (cl. 4–6) to pollen, wheat flour and hazelnut, with low IgG but high total IgE, IL-4, IL-8 and IFNγ	subjects with allergy symptoms, on elimination diet but not pharmacotherapy, with negative family history, born by caesarean section and formula or mixed fed, whose mothers took probiotics during pregnancy and stayed on gestational cholestatic diet	0.192
N	low or no specific IgE antibodies in serum, (cl. 1–2) to dust mites, beef and potato, with moderate IgG (10–20 g/l) and low total IgE and all tested cytokines	subjects pharmacologically treated, on elimination diet, with positive family history, with overweight BMI status, naturally born, breastfed and taking probiotics in infancy, whose mothers were on gestational cholestatic and diabetic diet and took probiotics	0.269
O	high (cl. 5–6) level of antibodies to milk, dander, nuts, soybean and pollen, with high IgG (>20 g/l) and high total IgE and all tested cytokines	subjects with positive allergy history and severe symptoms, naturally born, breastfed, whose mothers were on gestational cholestatic diet and took probiotics	0.213
P	moderate (cl. 2–4) level of antibodies to milk (β-Lg and BSA) and beef proteins, and moderate (cl. 3–4) to eggs with moderate (10–20 g/L) IgG but high total IgE and IL-4	subjects with positive allergy history, naturally born infant formula fed, on elimination diet and pharmacologically treated, whose mothers were on vegan/ vegetarian diet and took probiotics	0.229
Q	moderate (cl. 2–4) level of antibodies to milk (casein) and soybean proteins, and high (cl. 5–6) to nuts and mites with moderate (10–20 g/L) IgG but high total IgE, IL-4 and IL-8	subjects with positive allergy history, naturally born infant formula fed, on elimination diet and pharmacologically treated, whose mothers were on gestational cholestatic diet and took probiotics	0,236
R	healthy individuals with normal antibodies levels but high IL-4 and IL-8	with negative family history, born by caesarean section and were formula or mixed fed, whose mothers were vegan/vegetarian diet during pregnancy	0.248
S	high (cl. 5–6) to pollen, mites, nuts and dander, with low IgG but high total IgE, IL-4 and IL-8	subjects with allergy symptoms but untreated, with negative family history of allergy, born naturally and breastfeed,	0.260

	whose mothers took probiotics during pregnancy and eliminated milk
